# Iodixanol Has a Favourable Fibrinolytic Profile Compared to Iohexol in Cardiac Patients Undergoing Elective Angiography: A Double-Blind, Randomized, Parallel Group Study

**DOI:** 10.1371/journal.pone.0147196

**Published:** 2016-01-19

**Authors:** Andrew T. Treweeke, Benjamin H. Maskrey, Kirsty Hickson, John H. Miller, Stephen J. Leslie, Ian L. Megson

**Affiliations:** 1 Department of Diabetes & Cardiovascular Science, University of the Highlands & Islands, Inverness, United Kingdom; 2 Radiology, NHS Highland, Inverness, United Kingdom; 3 Cardiology, NHS Highland, Inverness, United Kingdom; 4 School of Nursing, Midwifery and Health, University of Stirling, Inverness, United Kingdom; Harvard Medical School, UNITED STATES

## Abstract

**Background:**

There is no consensus and a limited evidence base for choice of contrast agents (CA) in angiography. This study evaluated the impact of iohexol and iodixanol CA on fibrinolytic factors (tissue plasminogen activator [t-PA] and plasminogen activator inhibitor-1 [PAI-1]), as well as platelet-monocyte conjugates in cardiac patients undergoing elective angiography in a double-blind, randomised parallel group study.

**Methods:**

Patients (men, 50–70 years old; n = 12) were randomised to receive either iohexol (Omnipaque; n = 6) or iodixanol (Visipaque; n = 6) during elective angiography at Raigmore Hospital, Inverness, UK. Arterial and venous blood samples were drawn prior to CA delivery and following angiography. Assessment of platelet-monocyte conjugation, t-PA and PAI-1 antigen and activity was conducted in samples pre- and post-angiography.

**Outcome:**

Plasma t-PA antigen was depressed equally in the study groups after angiography, but there was a greater reduction in PAI-1 antigen in the group receiving iodixanol. These findings corresponded to a substantial reduction in t-PA activity in patients receiving iohexol, with no change in those receiving iodixanol (P = 0.023 between the CA groups). Both CAs caused a reduction in platelet-monocyte conjugation, with no difference between the groups. No adverse events were reported during the trial.

**Conclusion:**

Avoiding reduced plasma t-PA activity might be an important consideration in choosing iodixanol over iohexol in patients at risk of thrombosis following angiography. The trial is registered on the ISRCTN register (ISRCTN51509735) and funded by the Coronary Thrombosis Trust and National Health Service (Highland) R&D Endowments. The funders had no influence over study design or reporting.

**Trial Registration:**

Controlled-Trials.com ISRCTN51509735

## Introduction

There is considerable debate over the relative merits of the various contrast agents (CAs) used routinely in cardiology to enhance definition of coronary arteries for diagnostic angiography and for interventional techniques (angioplasty and stenting). Early research focused on comparisons of ionic (e.g. ioxaglate) and non-ionic (e.g. iohexol; Omnipaque^™^) CAs; iohexol was found to have a moderately better haemodynamic profile than ioxaglate [1]. Thrombogenicity is considered to be a risk of CA use, but the data are contradictory: non-ionic iopamidol was found to generate more thrombus than ionic diatrizoate in patients undergoing intervention procedures, but use of non-ionic iohexol during high risk percutaneous transluminal coronary angioplasty was associated with reduced incidence of major adverse cardiac events (MACE; 5.4%), compared to ioxaglate (9.5%) [2]. The notion that modulation of thrombotic potential is central to differential risk associated with CA [3] gave rise to a number of in vitro studies to determine the impact of CA on platelet function. However, the direct inhibitory effects of CA on platelet activation in vitro [4, 5] do not reflect the clinical data. Indeed, ioxaglate has been shown to have a powerful inhibitory effect on thrombus formation in vitro, while non-ionic CA actively induce formation of thrombus that is particularly resistant to thrombolysis [6]. Iohexol-induced resistance to thrombolysis might be compounded in vivo because iohexol, but not ioxaglate, increases plasminogen activator inhibitor 1 (PAI-1) [7], with the potential to reduce fibrinolysis and increase thrombotic risk—a finding that contradicts clinical outcomes [2].

More recently, another category of CA has emerged—the non-ionic dimers (e.g. iodixanol; Visipaque^™^). Evidence suggests that iodixanol has a favourable outcome profile in coronary angiography, not only compared to ionic ioxaglate in high-risk patients undergoing coronary angioplasty [2], but also compared to the non-ionic monomer, iohexol, especially in patients with unstable angina [8]. In vitro platelet studies do not provide a clear answer as to how the benefits of iodixanol are realised: thrombus formation has been found to be more substantial with this CA than with either iohexol or ioxaglate, but iohexol had the additional disadvantage of enhanced platelet degranulation [6]. However, other studies have shown a reduced impact of iodixanol on platelet activation than iohexol or diatrizoate [9] and a less detrimental effect in cultured endothelial cells than either iohexol or ioxaglate [10]. An impact of CA on the endothelium provides a particularly interesting route to thrombosis because endothelial cells can influence platelet function both through release of pro- and anti-platelet mediators (e.g. nitric oxide, prostacyclin, thromboxane A_2_) [11], and through generation of pro- and anti-fibrinolytic factors—tissue plasminogen activator (t-PA) [12] and plasminogen activator inhibitor 1 (PAI-1) [13], respectively. Endothelial function is also highly responsive to oxygen-centred free radicals (oxidative stress) and inflammation [11], both of which have been implicated in the aetiology of CA-induced nephrotoxicity.

Here, we tested the hypothesis that iodixanol, but not iohexol, influences the release of t-PA and PAI-1 to favour increased fibrinolysis in patients undergoing elective coronary angiography. In addition, we measured platelet-monocyte conjugates as a recognised surrogate for cardiovascular risk [14–19]. Given the well-recognised sensitivity of both the endothelium and platelets to oxidative stress, oxygen-centred free radical generation from iodixanol and iohexol was measured *in vitro* to assess the potential for oxidative stress to play a role in CA-induced thrombosis.

## Materials & Methods

### Ethics Statement

Ethical approval from the North of Scotland Research Ethics Committee (REC: 11/S0802/14, IRAS ID: 70700) was obtained in advance of the study (approval date 24/3/2011), which complied with the Declaration of Helsinki and its amendments. All patients gave written, informed consent. The formal study completion date was 1^st^ May 2013. The trial is registered on the ISRCTN register (ISRCTN51509735). Trial registration was retrospective because planning for the study began in 2010, before trial registration for small investigator led single site studies was the norm. The authors confirm that all ongoing and related trials for this intervention are registered. The ethics protocol is included in (S1 File).

### Patients

**Fig 1:**
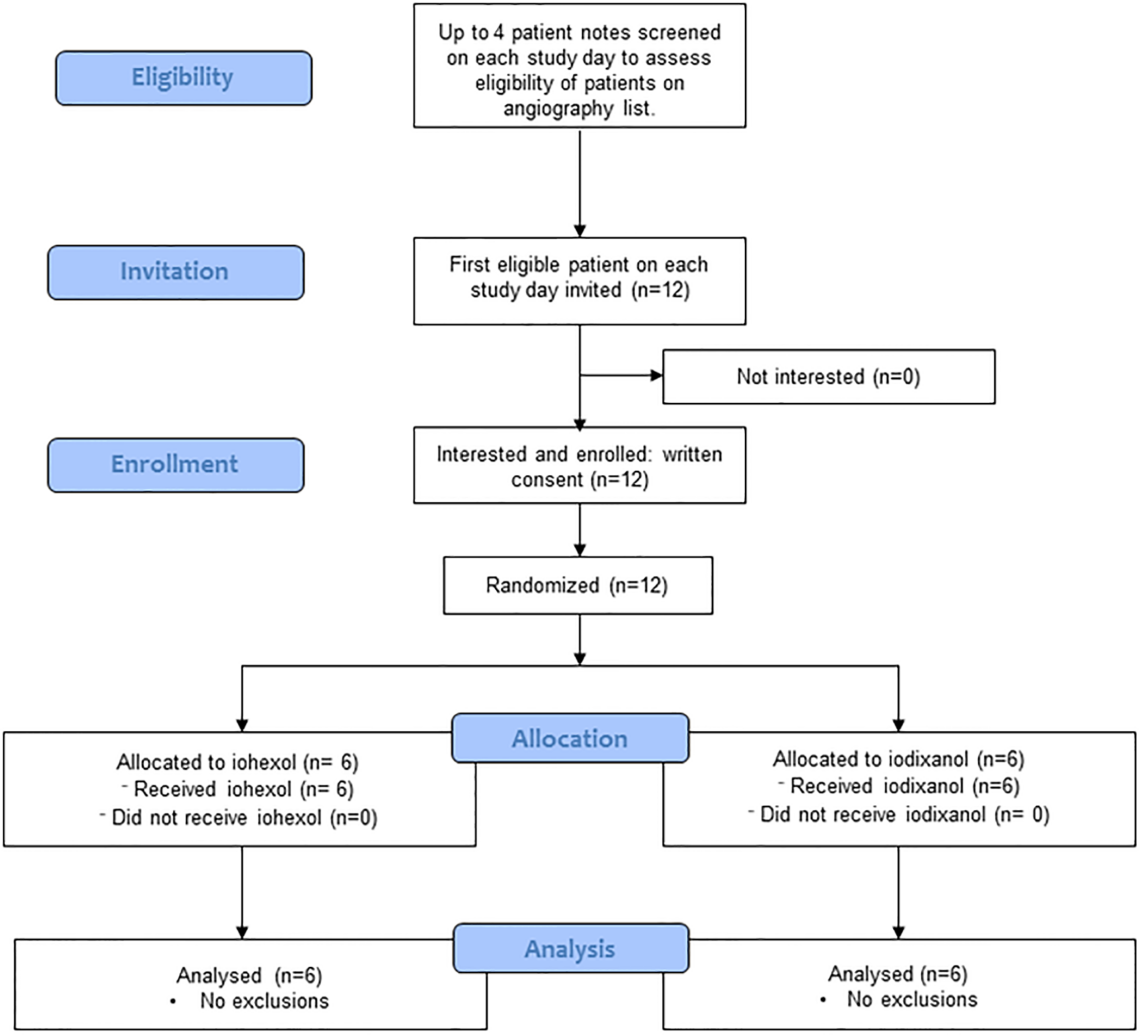
Flow diagram for patient recruitment, enrolment and participation in the study.

Informed consent was obtained from patients with coronary artery disease attending Raigmore Hospital, Inverness, UK between June and July 2011. Inclusion criteria for the study were: men, 50–70 years old, prescribed aspirin and scheduled for coronary angiography. Patients prescribed clopidogrel and those who had taken part in any study in the previous 3 months were excluded. Patients were requested to abstain from alcohol and caffeine 12 h prior to angiography and were randomised to receive either iohexol (Omnipaque^™^ 300 mg I/ml) or iodixanol (Visipaque^™^ 320 mg I/ml). The volume of CA received varied according to need during the procedure (minimum 40 mL, maximum 185 mL; see Table 1 for mean data). On each study day, notes from up to 4 patients on the clinical angiogram list for that morning were consulted to assess eligibility against the inclusion and exclusion criteria (Fig 1). The first patient on each day that was eligible for the study was invited to participate. All patients who were approached to participate in the study consented. Only one patient was recruited on each study day; only patients scheduled for morning angiograms were recruited to the study to minimise the impact of circadian variation on fibrinolytic [20, 21], platelet [22] and endothelial [23, 24] function. The Consort checklist is included in (S2 File).

**Table 1:** Patient characteristics.

	Iohexol (Omnipaque)	Iodixanol(Visipaque)
Patients recruited (n)	6	6
Evaluable patient number (n)	6	6
Age (yrs±SD)	65.5±6.5	67.5±7.4^NS^
BMI (±SD)	26.9±6.2	33.8±7.2^NS^
SBP (mmHg; ±SD)	154±24	146±11^NS^
DBP (mmHg; ±SD)	77±12	80±10^NS^
Hypertension (n)	2	2^NS^
Aspirin (n)	6	6^NS^
ACE inhibitors (n)	1	2^NS^
Ang II receptor antagonists (n)	1	1^NS^
β-blockers (n)	2	3^NS^
Calcium antagonists (n)	1	2^NS^
Statins (n)	5	6^NS^
Nitrates (n)	0	1^NS^
Contrast volume received (ml)	73±9	99±19^NS^

^NS^ Not statistically different from iohexol group (Student’s *t*-test for continuous data, χ^2^ test for categorical data).

### Power calculation, randomization, blinding

A power calculation was performed using the SD from published data from a related study with PAI-1 as an outcome measure [7], using Lehr’s formula. On the basis of the calculation, it was determined that 6 patients would be required in each group to give 80% power of detecting a 50% change in PAI-1 at the P<0.05 confidence level. Patients were randomized to receive either iohexol or iodixanol by a researcher independent to the study using a random number generator. Researchers and patients were blinded as to which CA was used–each patient was assigned a code number and none of the researchers had access to the key; unblinding took place after analysis of the data was complete for the whole study.

### Blood sampling and laboratory analyses

Blood (25 ml) was sampled from both 5F arterial sheath and 21G venous peripheral venous cannula (in the antecubital fossa) at baseline (immediately prior to CA injection) and immediately following angiography (the time between samples was typically ~20–30 min). At each draw, the first 10 ml of blood was discarded. An aliquot of samples was taken into lithium-heparin tubes (Monovette^®^) for flow cytometry assessment of platelet-monocyte conjugation, whilst a second aliquot was taken into citrate tubes (pH 4.3) prior to centrifugation (5000 *g*, 10 min) and isolation of plasma that was immediately frozen and stored in advance of batch-testing for t-PA and PAI-1 antigen and activity (Zymutest, Quadratech Diagnostics). Flow cytometry was carried out within 1 h of sampling: whole blood samples (50 μl) were dual-stained by incubating with CD14-FITC (BD 555397) and CD41a-PE-Cy5 (BD 559768) antibodies, or the appropriate isotype controls for 15 min. Samples were incubated for a further 15 min with FACS lysing solution (0.5 ml; BD) and then immediately analysed for conjugates using a FACSCalibur flow cytometer (Becton Dickinson). Platelet-monocyte conjugates were identified as CD14/CD41a–positive events [14].

### Protein Estimation

Plasma protein was estimated using Coomassie Plus protein assay reagent (Thermo Scientific). Briefly, citrated plasma samples were diluted 1:50 in PBS and 2 μl transferred to duplicate wells of a 96-well plate. To each well, 100 μl of Coomassie reagent was added, mixed and incubated for 10 min at room temperature before reading at λ = 595 nm using a Varioskan plate reader (Thermo). Sample protein values were estimated from the standard curve using the plate reader software.

### Oxygen-centred radical generation from iohexol and iodixanol (EPR spectrometry)

Electron paramagnetic resonance (EPR) spectrometry was used to establish the rate of generation of oxygen-centred radicals from iohexol and iodixanol. This in vitro assay involved dilution of CA to 80% v/v using phosphate-buffered saline (PBS) to facilitate uptake into capillary tubes (50 μl) prior to incubation with an oxygen-centred radical-specific spin trap (Tempone-H; 1 mM) at 37°C. Parallel experiments with and without vitamin C (200 μM) were conducted to examine the impact of an antioxidant on the signals generated. Samples were measured at 15 min intervals for 1 h in an EPR spectrometer (MS200 miniscope spectrometer; Magnettech, Germany; parameter settings: B0-field, 335.6mT; sweep width, 5 mT; sweep time, 30 sec; modulation amplitude, 0.15 mT; microwave power, 20 mW; microwave frequency, 9.3 GHz). The principle of the assay is that oxygen-centred radicals react with the spin trap to generate a spin-adduct (4-oxo-tempo), which is a stable free radical that generates a characteristic EPR spectrum [25] when excited with microwaves. The amplitude of the signal is directly proportional to the concentration of the spin adduct, allowing accurate comparisons of the rate of radical generation in the samples.

### Data and Statistics

**Fig 2:**
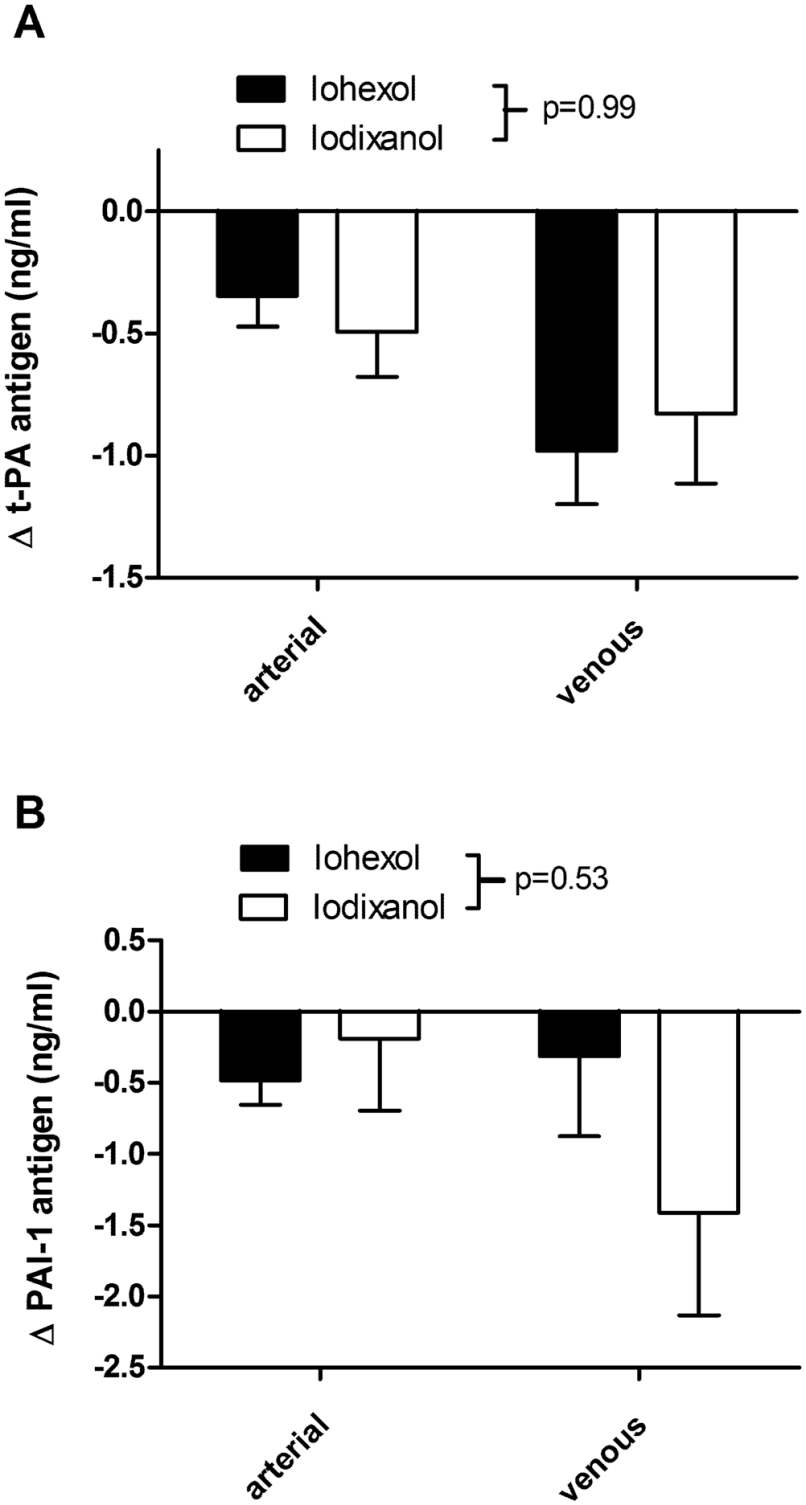
Change in (A) t-PA and (B) PAI-1 antigen in arterial and venous blood samples post-angiography compared to baseline in patients receiving iohexol and iodixanol. There was no significant difference between the groups (2-way repeated measures ANOVA, P value shown is between treatment groups; n = 6 in each group). There was no interaction between treatment groups and arterial/venous plasma.

**Fig 3:**
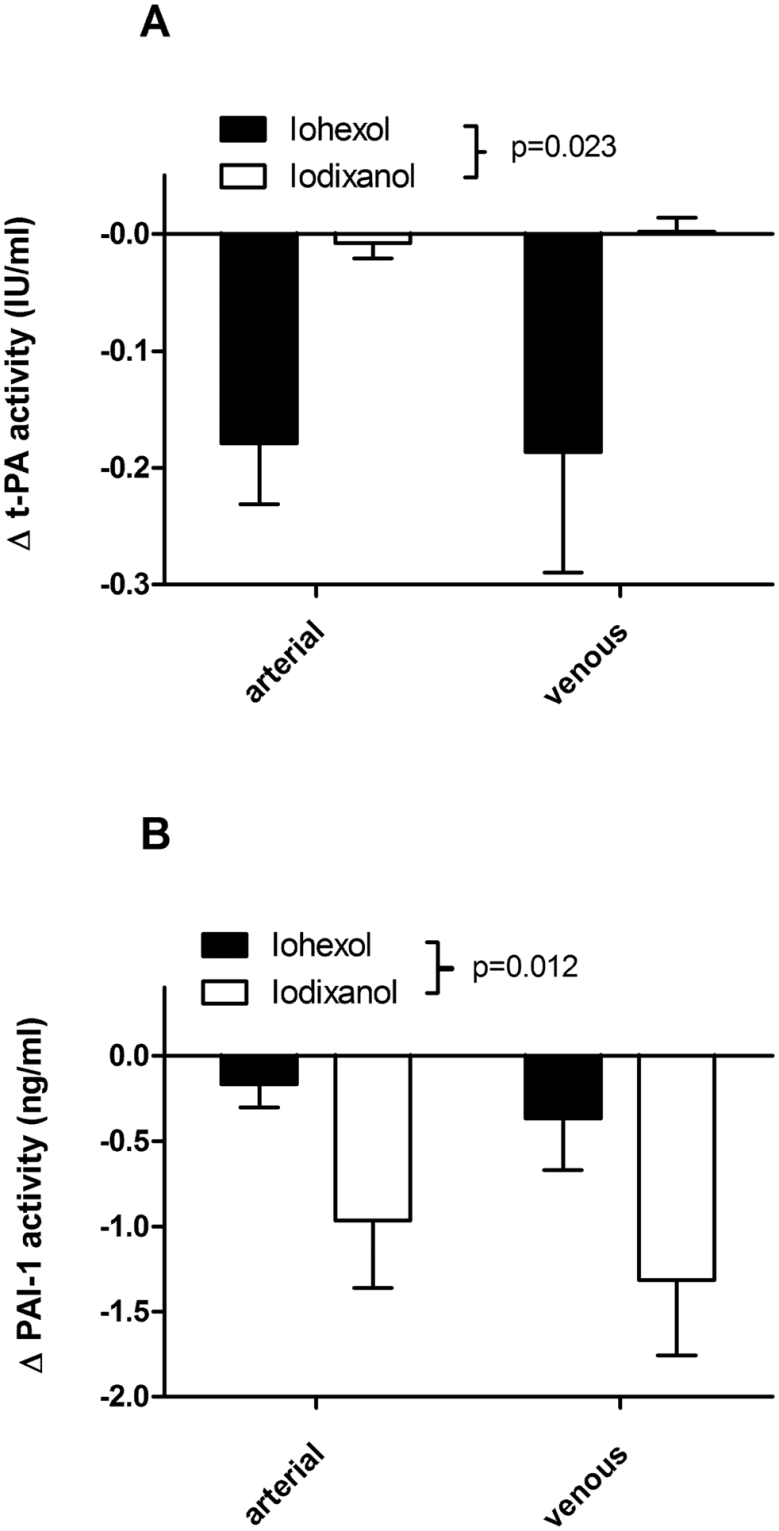
Change in (A) t-PA and (B) PAI-1 activity in arterial and venous blood samples post angiography compared to baseline in patients receiving iohexol and iodixanol. t-PA activity was significantly reduced in the iohexol group; PAI-1 activity was significantly reduced in the iodixanol group (2-way repeated measures ANOVA, P value shown is between treatment groups; n = 6 in each group). There was no interaction between treatment groups and arterial/venous plasma.

**Fig 4:**
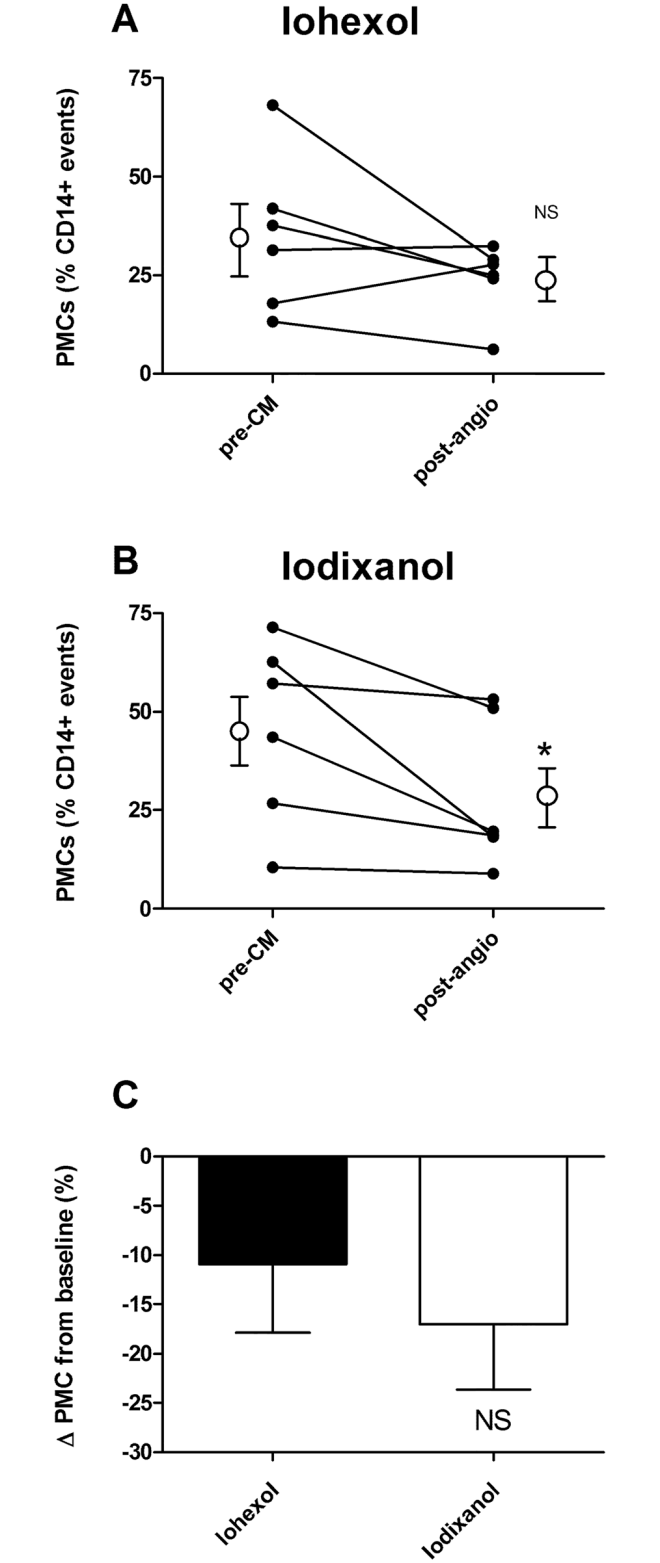
Effect of angiography (post-angio) on platelet-monocyte conjugation in patients exposed to (A) iohexol and (B) iodixanol compared to baseline (pre-CA); *P<0.05. The % reduction in platelet-monocyte conjugation is shown in (C). n = 6 in each group; NS—P>0.05.

**Fig 5:**
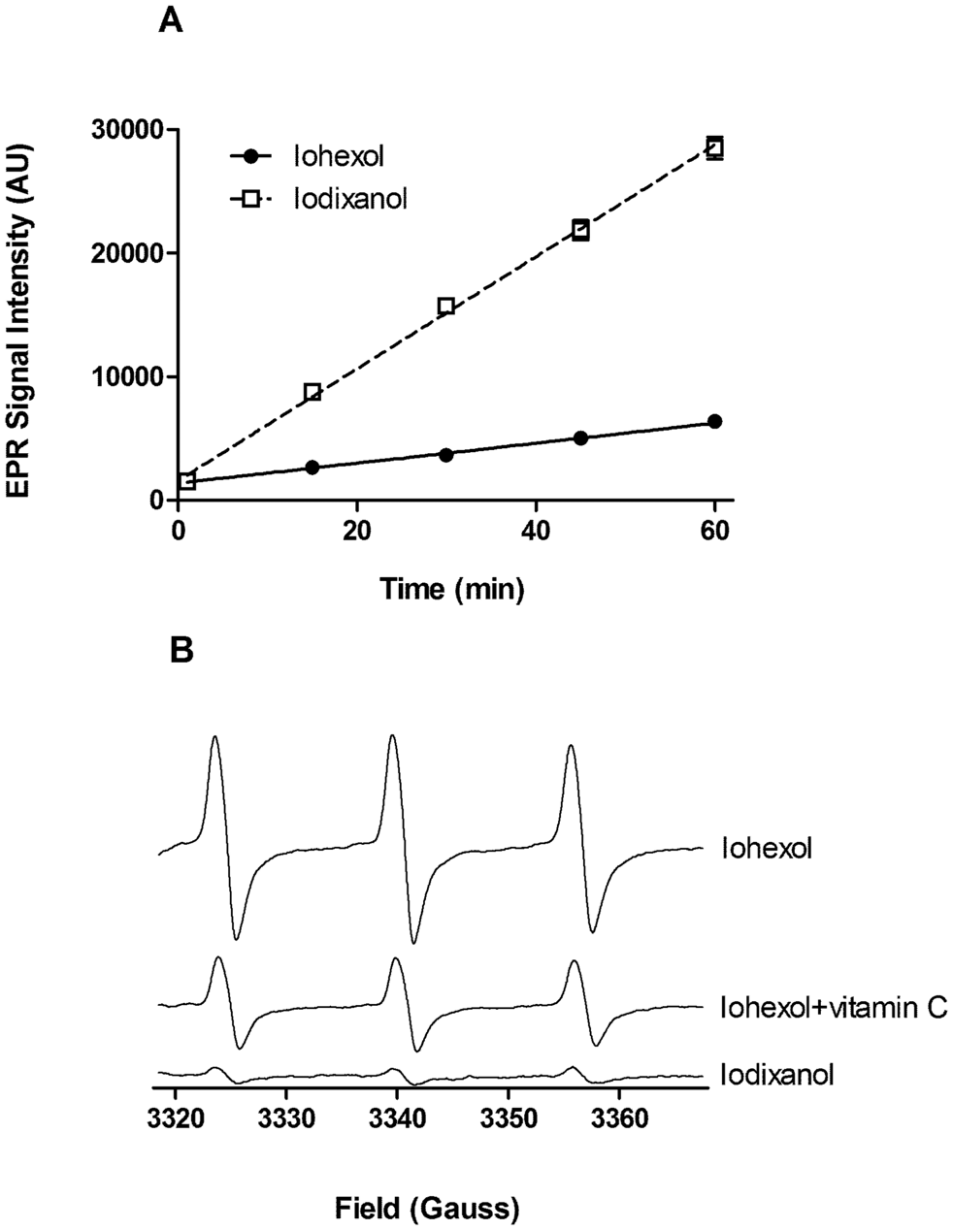
(A) *In vitro* oxygen-centred free radical generation in samples of CA at 37°C. Iohexol generates free radicals at a significantly higher rate than iodixanol (n = 4; P<0.001). (B) Representative EPR spectra obtained for iodixanol alone, iodixanol + vitamin C (200 μM) and iohexol after 60 min incubation (37°C) with the oxygen-centred radical spin trap, tempone-H (1 mM). The characteristic 3-line spectrum is representative of formation of the stable radical adduct, 4-oxo-tempone, via reaction with oxygen-centred radicals; the amplitude of the signal is proportional to the amount of 4-oxo-tempone present.

Data are presented as mean±SD (Table 1) and mean±SE (Figs 2–4). Patient characteristics (Table 1) and data in Fig 4 are compared with two-tailed, unpaired Student’s *t*-test (continuous data sets) and χ^2^ for categorical data sets. Data in Figs 2 and 3 are compared using 2-factor ANOVA (repeated measures) and those in Fig 5 using one-factor ANOVA. P<0.05 was accepted as significant in all cases.

## Results

Patient characteristics, pre-existing drug regimens and CA volume used are described in Table 1. No adverse events were reported in the trial. There was no significant dilution of plasma proteins in patients receiving either iohexol or iodixanol (S1 Table).

### t-PA and PAI-1 antigen and activity

Plasma t-PA antigen was reduced in both arterial and venous samples, but the extent of the reduction was smaller in arterial compared to venous samples, both in absolute and % baseline (~8% arterial, ~17% venous; S1 Table) terms. There was no statistical difference between the changes in plasma t-PA antigen following iohexol compared to iodixanol (Fig 2A).

Plasma PAI-1 antigen was also reduced in both arterial and venous samples in patients receiving iohexol or iodixanol. Although there was an apparent difference between the treatment groups with respect to PAI-1 antigen in the venous samples, this did not reach statistical significance (Fig 2B). The mean % reduction in PAI-1 antigen ranged from ~4–19% across the groups (S2 Table).

t-PA activity in the iodixanol group was relatively unaffected in arterial or venous samples post-angiogram, in keeping with equivalent reductions in both t-PA and PAI-1 antigen in this group. However, t-PA activity was significantly supressed in the group that received iohexol (Fig 3A), reflecting the imbalance between the reduction in t-PA antigen and that of PAI-1. The reverse was found for PAI-1 activity, where there was a significantly greater reduction in PAI-1 activity associated with the group that received iodixanol (Fig 3B). Raw data are available in (S3 Table).

### Platelet-monocyte conjugates

Platelet-monocyte conjugates were not significantly reduced in post-angiogram venous samples from patients receiving iohexol (Fig 4A), but this measure was significantly reduced in those receiving iodixanol (Fig 4B). There was no significant difference between the extents of reduction in platelet-monocyte conjugate counts in the two groups (Fig 4C). Raw data are available in (S4 Table).

### Oxygen-centred radical generation from iohexol and iodixanol

*In vitro* generation of oxygen-centred free radicals was measured in samples of iohexol and iodixanol incubated in the dark at 37°C. Iohexol was found to generate free radicals at a considerably faster (~x4) rate than iodixanol (Fig 5A). Fig 5B shows the reduction in EPR signal intensity generated by iohexol in the presence of the antioxidant, vitamin C (200 μM), and the very low signal intensity generated by iodixanol after 60 min incubation. Raw data are available in (S5 Table).

## Discussion

The choice of CA in the cardiology setting is founded on a limited evidence base and the merits of each are debated. This study examined the differences between two of the most commonly used CA, iohexol and iodixanol, on factors central to the fibrinolytic process and on platelet-monocyte conjugation as a marker of thrombotic risk, with a view to informing the debate over CA choice.

The key findings were that t-PA activity was reduced in patients receiving iohexol, but remained unchanged in those receiving iodixanol. Platelet-monocyte conjugation was substantially reduced following angiography, but there was no difference between the extent of conjugate reduction in the groups receiving different CAs. Iohexol also generated oxygen-centred free radicals at a substantially faster rate than iodixanol *in vitro*.

t-PA is a crucial endothelium-derived fibrinolytic factor that activates the conversion of plasminogen to plasmin. Activity of t-PA is modulated by PAI-1, a factor that is also generated by the endothelium as well as by activated platelets. PAI-1 conjugates with t-PA to inactivate it; the balance of these two factors is central to determining the extent of fibrinolysis. Ionic and non-ionic CAs differentially affect PAI-1; iohexol has been found to have a detrimental effect with respect to PAI-1 release compared to ioxaglate [7]. The current study investigated the newer non-ionic dimeric CA, iodixanol, to establish if it behaved in the same way as iohexol with respect to PAI-1 generation. Our findings indicate that iodixanol has little impact on the balance of t-PA to PAI-1, whereas iohexol causes a net reduction in t-PA activity. Although both iohexol and iodixanol reduce t-PA antigen, t-PA activity remains unchanged in the iodixanol group on account of equivalent modulation of PAI-1 release. Iohexol, on the other hand, induces a larger reduction in t-PA antigen than PAI-1 antigen, resulting in the measurable loss in t-PA activity. The effects seen were not confounded by circadian fluctuations [20, 21] because all angiograms were conducted in the morning and the pre- and post-angiogram samples were drawn only 20–30 min apart. That non-ionic iodixanol more closely aligns with ionic ioxaglate [7] than with non-ionic iohexolin in this regard suggests that the effects of CAs on fibrinolytic factors are not related to their ionic characteristics. Nor does it appear that the effects are mediated by a dilution effect of CA administration, given that plasma protein was not significantly affected by either CA. Instead, it seems that chemical characteristics specific to iohexol drive a net loss in t-PA activity on account of a mis-match between loss of t-PA antigen and PAI-1 antigen. The reduced PAI-1 activity in our study was at odds with the findings of a previous study [7], in which PAI-1 activity was found to increase with iohexol; the major difference between the two studies was the patient group (suspected pulmonary embolism as opposed to coronary artery disease) and it is plausible that fibrinoloyic factors could be differentially affected in these quite diverse patient groups. The impact of reduced t-PA activity with iodixanol *in vivo* would compound the recognised resistance of thrombi formed with non-ionic CA to thrombolysis *in vitro* [6]. These characteristics, coupled with a reduced propensity to platelet degranulation [6], might help to explain the improved outcome in patients receiving iodixanol compared to those receiving iohexol.

Platelet function has long been known to be affected by CAs, but this is the first study to measure platelet-monocyte conjugation as a marker of thrombotic potential. Exposure to both CAs reduced platelet-monocyte conjugates, suggesting an antithrombotic effect, although statistical significance was only reached for iodixanol in this small study. There was no significant difference between the extents of reduced platelet-monocyte conjugation between the two CAs tested. These findings are in agreement with previous data suggesting that CAs have antiplatelet effects *in vitro* [4, 5].

Taking into account both the platelet-monocyte conjugation and the fibrinolytic factor data, it can be surmised that CAs might reduce risk of thrombosis in the short term but that, should a thrombotic event occur, patients receiving iohexol might suffer more severe effects on account of depressed fibrinolytic defence. The findings shift the emphasis of CA-induced dysfunction away from platelets and towards the endothelium, as the principal source of t-PA and PAI-1, and the vasoactive factors that it generates.

Endothelial activity is highly sensitive to oxidative stress, so we explored whether there were differences in oxygen-centred free radicals generation from iohexol and iodixanol that might contribute to the effects seen. Previous findings suggest that CAs have an antioxidant effect overall and that iodixanol is more potent in this regard than iohexol [26], but the current study indicates that iohexol induces substantial spontaneous oxygen-centred free radical formation compared to iodixanol; the radical production is partially quenched by a moderate concentration of the antioxidant, vitamin C, confirming that the signal is mediated by oxygen-centred radical generation.

At present, we have no evidence to link the free radical generation seen with iohexol to endothelial dysfunction or to the impact on PAI-1 and t-PA. However, the current data could provide an explanation as to why there are differential effects of these CAs on release of fibrinolytic factors. The hypothesis that oxygen-centred radical generation from iohexol drives endothelial dysfunction with respect to release of fibrinolytic factors is worthy of further investigation.

### Study limitations

This was a small, but adequately powered study for the primary outcomes. In addition, the study was only conducted in men undergoing elective angiography. A large, multi-centre trial is justified to determine whether the results are reproduced at scale.

## Conclusions

Iohexol and iodixanol both induced an inhibitory effect on platelet-monocyte conjugation, suggestive of an anti-thrombotic effect of both CAs. However, iohexol also induced a significant reduction in t-PA activity compared to iodixanol, implying that fibrinolytic capability is impaired after use of this agent. These findings contribute to the debate over which CA to use and justify a larger outcome study to establish whether the physiological changes reflect in major adverse coronary events, which could help cardiologists to make fully informed decisions regarding which CA to use in clinical practice.

## Supporting Information

S1 File Ethics Protocol. (PDF)

S2 File Consort checklist. (PDF)

S1 Table Data for Pre- and post-angioplasty measures, absolute change and % baseline change for all parameters measured (mean±SE for pre-and–post angioplasty data and absolute change; all normally distributed, as assess by Kolmogorov-Smirnov test; median (interquartile range) for % change, which was not consistently normally distributed). (DOCX)

S2 Table Raw data and statistical analyses associated with Fig 2. (PDF)

S3 Table Raw data and statistical analyses associated with Fig 3. (PDF)

S4 Table Raw data and statistical analyses associated with Fig 4. (PDF)

S5 Table Raw data and statistical analyses associated with Fig 5. (PDF)

## Abbreviations

ANOVA: analysis of variance
CA: contrast agent
EPR: electron paramagnetic resonance
MACE: major adverse cardiac events
PAI-1: plasminogen activator inhibitor-1
SD: standard deviation
SE: standard error of the mean
t-PA: tissue-type plasminogen activator
