# Supplementary material for: Iodixanol Has a Favourable Fibrinolytic Profile Compared to Iohexol in Cardiac Patients Undergoing Elective Angiography: A Double-Blind, Randomized, Parallel Group Study
Source: PLoS One. 2016 Jan 19;11(1):e0147196. doi: 10.1371/journal.pone.0147196 (PMC4718690; doi:10.1371/journal.pone.0147196)
Supplement: S2 File — (PDF) [file pone.0147196.s002.pdf]

Full Title: A pilot study into the effects of intravascular contrast media on platelet function in patients undergoing coronary angiography

Short title: The effects of contrast media on platelet function

Investigators:

[1]Andrew Treweeke Researcher

[1,2]Stephen J Leslie Consultant Cardiologist

[1]Ian L Megson Professor

Affiliations:

[1]Department of Diabetes and Cardiovascular Science, UHI Millennium Institute, Centre for Health Science. Inverness

[2]Cardiac Unit, Raigmore Hospital, Old Perth Road, Inverness, UK. IV2 3UJ

Background and purpose of research

Iodinated contrast media (CM) are used routinely in cardiology to enhance definition of coronary arteries for diagnostic angiography and for interventional techniques (angioplasty and stenting). Several CM preparations are commercially available: all contain covalently bound iodine and they are broadly categorized according to whether they are ionic (e.g. ioxaglate) or non-ionic (e.g. iodixanol and iohexol). From an osmolarity perspective, the consensus is that *non-ionic* CM are advantageous over their ionic counterparts, on account of their lower osmolarity, a characteristic that was originally believed to contribute to a decrease in incidence of major complications associated with diagnostic cardiac catheterization.[1] This finding has since been disputed in large-scale studies[2] and in vitro testing suggests that *ionic* CM have properties that are less likely to induce thrombotic events because they have more pronounced anticoagulant activity, they do not activate resting platelets[3a] and they are less inclined to potentiate the actions of recognised endogenous platelet agonists (ADP and platelet activating factor) and even to actively inhibit thrombin-induced aggregation.[3] Indeed, there is little evidence to confirm that a direct platelet action is the cause of the detrimental findings in the clinical setting, save for reports of increased guidewire platelet deposition.[4] Equally, whilst there is some evidence to support a role for CM in platelet degranulation,[5] the underlying mechanism by which CM might cause enhanced sensitivity to conventional agonists is not known, although it appears to involve changes in calcium signalling.

Given that the platelet effects of CM in vitro are well-recognised, it is unsurprising that an association between the use and type of CM with thrombus-related events has been proposed. Data from a multi-centre trial of ionic versus non-ionic contrast agents in high risk percutaneous transluminal coronary angioplasty indicated that use of the non-ionic CM, iodixanol, was associated with an incidence of only 5.4% of major adverse clinical events, compared to 9.5% for the ionic CM, ioxaglate.[6] The propensity of CM to induce non-Q wave myocardial infarction might also suggest that any adverse effect is either transient or relatively

weak in nature. However, these and other similar findings have had little apparent impact on clinical practice with respect to choice of CM in practice.

The research to date in this field has been largely observational, leading to an overall perception that choice of CM might have an impact on clinical outcome, but the conflicting results regarding possible platelet effects perhaps suggests that thrombogenicity of CM is not through a direct platelet activating effect. Indeed, there is separate evidence to suggest that CM has a rapid adverse effect on endothelial cell morphology,[7] which might indirectly contribute to thrombus formation via loss of endothelium-derived protective agents (e.g. nitric oxide; NO and prostacyclin). Circumstantial evidence surrounding the rapidity of onset of events (generally intra-procedural), would suggest that adverse effects are unlikely to be mediated via induction of complex pathways that require modulation of gene transcription (e.g. inflammation). Instead, direct chemical interaction is far more likely to account for the effects and oxidative inactivation of the ordinarily protective free radical, NO, is a prime candidate.

Study aims

The aims of this study are two-fold:

1. to obtain pilot flow cytometry data to establish whether platelets from patients undergoing angiography or interventional cardiology procedures are activated and to confirm whether any difference in activation is evident with ionic vs non-ionic CM.

2. to test the hypothesis that inactivation of NO by oxidative stress is a factor in the pro-thrombotic state associated with CM through induction of acute endothelial dysfunction.

Plan of Investigation

1. ***In vitro assessment of the interaction of CM with nitric oxide: role of oxidative stress***

The possibility of chemical inhibition of NO, which is ordinarily generated by the endothelium and platelets, will be assessed by testing the quenching effect of CM on NO from a donor drug (DETA/NO). The oxygen-centred radical generating capacity of ionic and non-ionic CM will be assessed using electron paramagnetic resonance (EPR) spectrometry. Briefly, this involves treating samples with a spin trap that is specific for oxygen-centred radicals and measuring the formation of the stable spin-adduct that is generated in the presence of oxygen-centred radicals with time. In the event that one or more of the CM are found to generate significant radicals, inclusion of simple antioxidants (e.g. vitamin C) in the medium will be assessed in vitro.

2. ***Ex vivo analysis of platelet function and thrombotic risk***

To our knowledge, flow cytometry has not yet been applied to the question of thrombotic risk associated with CM. This technique allows assessment of platelet activation ex vivo (e.g. through measurement of surface P-selectin exposure) in the immediate aftermath of CM infusion and is as close as we can yet get to an in vivo measure of platelet activation. The technique also facilitates simultaneous measurement of platelet-leucocyte adhesion, recently identified as a marker for risk of MI.[8] We will assess both of these measures in blood from patients undergoing routine angiography. The frequency of procedures at Raigmore Hospital will enable us to have rigorous selection criteria for this study: inclusion criteria will include gender (male) aged 50-70 years old and exclusion criteria will include anti-platelet agents other than aspirin. The study will be of double-blind, randomised design for non-ionic and ionic CM (n=6 for each). Coronary arterial blood will be drawn immediately before, during and 5 min after an infusion of CM and taken for immediate assessment of platelet function and cellular oxidative stress using flow cytometry (P-selectin

analysis, platelet-monocyte binding, oxidative stress markers). Plasma samples will also be assessed for tissue plasminogen activator (t-PA) and plasminogen activator inhibitor (PAI-1) to help identify whether there is any effect of ICM on fibrinolytic capacity.

Outcomes and Impact

Despite observational studies that highlight the issue of CM choice as an important factor in risk of major adverse events associated with angiography and interventional procedures, there is considerable confusion over the mechanism that underpins the effect and the reason for improved outcome with ionic CM. This pilot study will help to establish a causal link between platelet activation and exposure to CM in vivo and will turn the spotlight on impaired endothelial function as a possible trigger for both vasospasm and increased platelet adhesion and aggregation. The techniques employed will particularly enable us to identify the role of oxidative damage in platelets and endothelial cells and will help point the way to possible solutions to the problem.

Study design

<u>Patients with coronary artery disease</u>

Patients (n=12) who are scheduled for coronary angiography will be recruited from the cardiology clinic and will be asked to abstain from alcohol and caffeine 12 hr prior to blood sampling.

Inclusion criteria

Male

Aged 50-70

Clinical reason for coronary angiography

Coronary artery disease defined by coronary angiography

No history of diabetes, Fasting Blood glucose < 6 mmol/l, HbA1c < 6.5%

Taking aspirin but not clopidogrel

Non-smoker

Able to give informed consent

Exclusion criteria

Participation in pharmacological study within last 3 months

Blood sampling

Blood will be sampled from both the arterial and venous circulation; 25 ml of blood will be required at each time point and site, therefore requiring 100ml in total. Upon arrival in the laboratory, an aliquot of whole blood will be treated with specific antibodies for detection of platelet-monocyte conjugates. A further aliquot will be centrifuged (~180*g*) to generate platelet rich plasma for measurement of platelet P-selectin. Finally, the remaining blood will be centrifuged to remove all cells and 3ml of the resulting plasma centrifuged at 16000g to purify microparticles for flow cytometric studies. The remaining plasma will be frozen for assessment of changes in fibrinolytic activity.

Power calculation

This is a pilot project and thus performing an accurate power calculation is not possible. From our own previous data using this technique. For a significance level of 5% and a power of 80% for detecting a change in aggregation of 15% in paired samples (patient group vs control):

Standardised difference = 2 x (15/27.9) = 1.075

Using Lehr's Formula: n= $16/1.075^2$ = **13.8**

However, is is unknown what the effect of contrast media will be on plaetelt function and therefore for the purposes of this pilot study we propose to study samples from 6 patients in each group.

Statistical analysis

Data will be entered into a spreadsheet for further analysis. Dose response curves will be created and differences tested by 2 way ANOVA. Significance will be taken at the 5% level.

Key References

1. Bettman MA, Higgins CB. Comparison of an ionic with a non-ionic contrast agent for cardiac angiography: results of a multicenter trial. *Invest Radiol.* 1985;20:570-574.
2. Davidson CJ, Mark DB, Pieper KS, Kuisslo KB, Hlatky MA, Gabriel DA, Bashore TM. Thrombotic and cardiovascular complications related to non-ionic contrast media during cardiac catheterization; analysis of 8,517 patients. *Am J Cardiol* 1990; 65:1481-1484.
3. Chronos NAF, Goodall AH, Wilson DJ et al. Profound platelet degranulation is an important side-effect of some types of contrast media used in interventional cardiology. *Circulation* 1993;88:2035-2044.
4. Gasperetti CM, Feldman MD, Burwell LR, Angello DA, Haugh KH, Owen RM, Powers ER. Influence of contrast media on thrombus formation during coronary angioplasty. *J Am Coll Cardiol* 1991;18:443-450.
5. Grabowski EF, Jang IV, Gold H, Head C, Benoit SE, Michelson AD. Variability of platelet degranulation by different contrast media. *Acad Radiol* 1996; S485-S487.
6. Davidson CJ, Laskey WK, Hermiller JB, Harrison JK, Matthai W, Vliestra RE, Brinker JA, Kereiakes DJ, Muhlestein JB, Lansky A, Popma JJ, Buchbinder M, Hirshfield JW. Randomized trial of contrast media utilization in high risk PTCA. *Circulation* 2000;101:2127-2177.
7. Barstad RM, Buchmann MS, Hamers MJ, Orning L, Orvim U, Stormorken H, Sakariassen KS. Effects of ionic and nonionic contrast media on endothelium and on arterial thrombus formation. *Acta Radiol.* 1996 37:954-61.
8. Sarma J. Laan CA, Alam S. Jha A. Fox KAA, Dransfield I. Increased platelet binding to circulating monocytes in acute coronary syndromes. *Circulation* 2002;105:2166-2171.
