# Supplementary material for: Iodixanol Has a Favourable Fibrinolytic Profile Compared to Iohexol in Cardiac Patients Undergoing Elective Angiography: A Double-Blind, Randomized, Parallel Group Study
Source: PLoS One. 2016 Jan 19;11(1):e0147196. doi: 10.1371/journal.pone.0147196 (PMC4718690; doi:10.1371/journal.pone.0147196)
Supplement: S1 Table — (DOCX) [file pone.0147196.s003.docx]

**S1 Supplementary Table**

| **Measure** | **Arterial/**  **Venous** | **Contrast agent** | **Pre-angioplasty** | **Post-angioplasty** | **Change (absolute)** | **Change**  **(% baseline)** |
| --- | --- | --- | --- | --- | --- | --- |
| **t-PA antigen**  **(ng/ml)** | **arterial** | iohexol | 4.59±0.70 | 4.24±0.74 | -0.35±0.13 | -8.9 (13.5) |
|  |  | iodixanol | 7.71±0.72 | 7.22±0.74 | -0.49±0.17 | -6.5 (7.9) |
|  | **venous** | iohexol | 5.65±0.86 | 4.68±0.72 | -0.98±0.20 | -18.4 (10.9) |
|  |  | iodixanol | 9.60±0.68 | 8.76±0.81 | -0.83±0.26 | -9.1 (11.8) |
| **t-PA activity**  **(IU/ml)** | **arterial** | iohexol | 0.47±0.22 | 0.29±0.14 | -0.18±0.05 | -40.9 (24.9) |
|  |  | iodixanol | 0.13±0.12 | 0.12±0.12 | -0.01±0.01 | -2.9 (14.5) |
|  | **venous** | iohexol | 0.60±0.12 | 0.41±0.11 | -0.19±0.09 | -11.7 (44.9) |
|  |  | iodixanol | 0.12±0.06 | 0.13±0.06 | +0.01±0.01 | 0.0 (6.3) |
| **PAI-1 antigen**  **(ng/ml)** | **arterial** | iohexol | 4.16±1.78 | 3.67±1.68 | -0.49±0.15 | -14.2 (16.4) |
|  |  | iodixanol | 12.17±1.54 | 11.98±1.89 | -0.19±0.51 | -4.5 (10.3) |
|  | **venous** | iohexol | 2.24±1.62 | 1.88±1.18 | -0.36±0.46 | -18.9 (46.5) |
|  |  | iodixanol | 9.59±1.16 | 8.18±1.36 | -1.41±0.66 | -8.9 (3.9) |
| **PAI-1 activity**  **(ng/ml)** | **arterial** | iohexol | 1.37±0.93 | 1.20±0.80 | -0.17±0.12 | -7.5 (11.5) |
|  |  | iodixanol | 7.83±1.65 | 6.86±1.65 | -0.96±0.36 | -10.5 (20.1) |
|  | **venous** | iohexol | 1.48±1.02 | 1.11±0.73 | -0.37±0.28 | -14.8 (16.9) |
|  |  | iodixanol | 7.53±1.54 | 6.21±1.53 | -1.32±0.40 | -19.8 (19.4) |
| **Platelet-monocyte conjugates (%)** | **arterial** | iohexol | 33.1±4.2 | 23.5±3.8 | -9.6±7.0 | -15.8 (47.6) |
|  |  | iodixanol | 28.2±7.2 | 36.2±10.4 | +8.0±5.5 | +31.0 (68.6) |
|  | **venous** | iohexol | 35.0±8.0 | 24.1±3.8 | -10.9±6.9 | -37.9 (44.3) |
|  |  | iodixanol | 45.3±9.4 | 28.2±7.7 | -17.1±6.6 | -29.5 (30.2) |
| **Plasma proteins**  **(mg/ml)** | **arterial** | iohexol | 23.4±0.9 | 24.8±0.77 | +1.5±1.4 | +3.4 (4.5) |
|  |  | iodixanol | 26.0±1.0 | 24.4±0.36 | -1.6±1.3 | -1.9 (6.5) |
|  | **venous** | iohexol | 25.3±1.6 | 22.9±1.2 | -3.3±1.4 | -13.4 (14.1) |
|  |  | iodixanol | 26.1±0.8 | 23.9±1.0 | -2.2±1.0 | -7.1 (16.0) |
