# Supplementary material for: Iodixanol Has a Favourable Fibrinolytic Profile Compared to Iohexol in Cardiac Patients Undergoing Elective Angiography: A Double-Blind, Randomized, Parallel Group Study
Source: PLoS One. 2016 Jan 19;11(1):e0147196. doi: 10.1371/journal.pone.0147196 (PMC4718690; doi:10.1371/journal.pone.0147196)
Supplement: S2 Table — (PDF) [file pone.0147196.s004.pdf]

S2 Original data for Fig 2

Fig 2A

Δ t-PA antigen (ng/ml)

	iohexol							
**subject**	1	2	3	7	11	12	**mean**	**SE**
**arterial**	-0.28	-0.02	-0.73	-0.49	-0.59	0.03	**-0.35**	**0.12**
**venous**	-1.03	-1.35	-0.54	-0.8	-1.81	-0.35	**-0.98**	**0.20**

	iodixanol							
**subject**	4	5	6	8	9	10	**mean**	**SE**
**arterial**	-0.45	-0.9	0.18	-1.05	-0.55	-0.19	**-0.49**	**0.17**
**venous**	-0.11	-0.9	-0.18	-1.34	-1.91	-0.54	**-0.83**	**0.26**

Table Analyzed	**t-PA antigen iohexol v iodixanol (Fig 2A)**				
Two-way RM ANOVA	Matching by rows				
Source of Variation	% of total variation		P value		
Interaction		1.9	0.3884		
art v ven		20.29	0.0825		
**Contrast agent**		**0**	**0.9921**		
Subjects (matching)		54.4746	0.0987		
Source of Variation	P value summary		Significant?		
Interaction	ns		No		
art v ven	ns		No		
**contrast agent**	ns		No		
Subjects (matching)	ns		No		
Source of Variation	Df		Sum-of-squares	Mean square	F
Interaction		1	0.132	0.132	0.8131
art v ven		1	1.411	1.411	3.724
**contrast agent**		1	0.00001667	0.00001667	0.000103
Subjects (matching)		10	3.79	0.379	2.334
Residual		10	1.624	0.1624	
Number of missing values		0			
Bonferroni posttests					
iohexol vs iodixanol					
art v ven	iohexol		iodixanol	Difference	95% CI of diff.
arterial		-0.3467	-0.4933	-0.1467	-0.7594 to 0.4660
venous		-0.98	-0.83	0.15	-0.4627 to 0.7627
art v ven	Difference		t	P value	Summary
arterial		-0.1467	0.6305	P > 0.05	ns
venous		0.15	0.6448	P > 0.05	ns

Fig 2B

Δ PAI-1 antigen (ng/ml)

	iohexol							
**subject**	1	2	3	7	11	12	**mean**	**SE**
**arterial**	-1.05	-0.78	-0.64	-0.3	-0.21	0.07	**-0.49**	**0.15**
**venous**	-0.22	-2.66	-0.21	0	-0.43	1.65	**-0.31**	**0.51**

	iodixanol							
**subject**	4	5	6	8	9	10	**mean**	**SE**
**arterial**	-0.61	-1.33	-0.86	-0.67	2.13	0.21	**-0.19**	**0.46**
**venous**	-0.21	-4.94	-0.53	-1.25	-1.01	-0.52	**-1.41**	**0.66**

Table Analyzed	**PAI-1 antigen iohexol v iodixanol (Fig 2B)**				
Two-way RM ANOVA	Matching by cols				
Source of Variation	% of total variation		P value		
Interaction		7.44	0.1339		
A v V		4.2	0.2483		
**contrast agent**		**2.46**	**0.5295**		
Subjects (matching)		57.9318	0.1332		
Source of Variation	P value summary		Significant?		
Interaction	ns		No		
A v V	ns		No		
treatment	ns		No		
Subjects (matching)	ns		No		
Source of Variation	Df		Sum-of-squares	Mean square	F
Interaction		1	2.919	2.919	2.661
A v V		1	1.649	1.649	1.503
contrast agent		1	0.964	0.964	0.4242
Subjects (matching)		10	22.72	2.272	2.072
Residual		10	10.97	1.097	
Number of missing values		0			
Bonferroni posttests					
iohexol v iodixanol					
treatment	iohexol		iodixanol	Difference	95% CI of diff.
arterial		-0.485	-0.1883	0.2967	-1.519 to 2.112
venous		-0.3117	-1.41	-1.098	-2.914 to 0.7174
treatment	Difference		t	P value	Summary
arterial		0.2967	0.3959	P > 0.05	ns
venous		-1.098	1.466	P > 0.05	ns
