# Supplementary material for: Iodixanol Has a Favourable Fibrinolytic Profile Compared to Iohexol in Cardiac Patients Undergoing Elective Angiography: A Double-Blind, Randomized, Parallel Group Study
Source: PLoS One. 2016 Jan 19;11(1):e0147196. doi: 10.1371/journal.pone.0147196 (PMC4718690; doi:10.1371/journal.pone.0147196)
Supplement: S3 Table — (PDF) [file pone.0147196.s005.pdf]

S3 Tables

Δ t-PA activity (IU/ml)

Fig 3A

	iohexol							
**subject**	1	2	3	7	11	12	**mean**	**SE**
**arterial**	-0.36	0.01	-0.13	-0.22	-0.26	-0.11	**-0.18**	**0.05**
**venous**	-0.02	0.02	-0.09	-0.47	-0.55	-0.02	**-0.19**	**0.09**

	iodixanol							
**subject**	4	5	6	8	9	10	**mean**	**SE**
**arterial**	-0.01	0.00	0.00	-0.05	0.04	-0.03	**-0.01**	**0.01**
**venous**	-0.05	0.00	0.00	0.00	0.03	0.04	**0.00**	**0.01**

Table Analyzed	**t-PA activity iohexol v iodixanol**			
Two-way RM ANOVA	Matching by cols			
Source of Variation	% of total variation	P value		
Interaction	0.07	0.862		
arterial vs venous	0	0.984		
**contrast agent**	**32.24**	**0.023**		
Subjects (matching)	44.6337	0.156		
Source of Variation	P value summary	Significant?		
Interaction	ns	No		
arterial vs venous	ns	No		
contrast agent	*	Yes		
Subjects (matching)	ns	No		
Source of Variation	Df	Sum-of-squares	Mean square	F
Interaction	1	0.0004431	0.0004431	0.03185
arterial vs venous	1	0.000006213	0.000006213	0.000447
contrast agent	1	0.1946	0.1946	7.223
Subjects (matching)	10	0.2694	0.02694	1.936
Residual	10	0.1391	0.01391	
Number of missing values	0			
Bonferroni posttests				
iohexol vs iodixanol				
contrast agent	iohexol	iodixanol	Difference	95% CI of diff.
arterial	-0.1791	-0.007596	0.1715	-0.02845 to 0.3714
venous	-0.1867	0.002015	0.1887	-0.01126 to 0.3886
contrast agent	Difference	t	P value	Summary
arterial	0.1715	2.078	P > 0.05	ns
venous	0.1887	2.287	P > 0.05	ns

Δ PAI-1 activity (ng/ml)

Fig 3B

	iohexol							
**subject**	1	2	3	7	11	12	**mean**	**SE**
**arterial**	-0.06	-0.84	-0.09	0.03	-0.05	0	**-0.17**	**0.12**
**venous**	-0.09	-1.86	-0.09	-0.24	-0.05	0.12	**-0.37**	**0.28**

	iodixanol							
**subject**	4	5	6	8	9	10	**mean**	**SE**
**arterial**	-1.33	-0.58	-1.99	-2.02	0.32	-0.19	**-0.97**	**0.36**
**venous**	-0.78	-1.59	-1.27	-0.37	-3.32	-0.56	**-1.32**	**0.40**

Table Analyzed	**PAI-1 activity iohexol v iodixanol**			
Two-way RM ANOVA	Matching by cols			
Source of Variation	% of total variation	P value		
Interaction	0.18	0.851		
arterial v venous	2.4	0.495		
contrast agent	24.15	0.012		
Subjects (matching)	25.3166	0.836		
Source of Variation	P value summary	Significant?		
Interaction	ns	No		
arterial v venous	ns	No		
contrast agent	*	Yes		
Subjects (matching)	ns	No		
Source of Variation	Df	Sum-of-squares	Mean square	F
Interaction	1	0.03375	0.03375	0.03729
arterial v venous	1	0.4538	0.4538	0.5013
contrast agent	1	4.559	4.559	9.539
Subjects (matching)	10	4.779	0.4779	0.528
Residual	10	9.052	0.9052	
Number of missing values	0			
Bonferroni posttests				
iohexol vs iodixanol				
media	omnipaque	visipaque	Difference	95% CI of diff.
arterial	-0.1683	-0.965	-0.7967	-1.960 to 0.3667
venous	-0.3683	-1.315	-0.9467	-2.110 to 0.2167
media	Difference	t	P value	Summary
arterial	-0.7967	1.659	P > 0.05	ns
venous	-0.9467	1.972	P > 0.05	ns
