# Supplementary material for: Iodixanol Has a Favourable Fibrinolytic Profile Compared to Iohexol in Cardiac Patients Undergoing Elective Angiography: A Double-Blind, Randomized, Parallel Group Study
Source: PLoS One. 2016 Jan 19;11(1):e0147196. doi: 10.1371/journal.pone.0147196 (PMC4718690; doi:10.1371/journal.pone.0147196)
Supplement: S4 Table — (PDF) [file pone.0147196.s006.pdf]

S4 Tables

Fig 4A

venous PMC data (CD14+ events (%))

iohexol			
subject	pre	post	
1	68.2	29	
2	37.6	25	
3	17.9	27.8	
7	41.9	24.2	
11	13.2	6.2	
12	31.4	32.4	
**mean**	**35.0**	**24.1**	
**SE**	**8.0**	**3.8**	
**paired t-test**	**P=**	**0.175**	NS

Fig 4B

venous PMC data (CD14+ events (%))

iodixanol			
subject	pre	post	
4	71.4	50.9	
5	62.5	18.2	
6	10.5	8.9	
8	43.5	19.6	
9	57.1	53.1	
10	26.7	18.6	
**mean**	**45.3**	**28.2**	
**SE**	**9.4**	**7.7**	
**paired t-test**	**P=**	**0.048**	*

Fig 4C

venous PMC data (ΔCD14+ events %)

iohexol			iodixanol		
subject	Δ PMC (%)		subject	Δ PMC (%)	
1	-39.2		4	-20.5	
2	-12.6		5	-44.3	
3	9.9		6	-1.6	
7	-17.7		8	-23.9	
11	-7.0		9	-4.0	
12	1.0		10	-8.1	
**mean**	**-10.9**			**-17.1**	
**SE**	**6.9**			**6.6**	
		**unpaired t-test**	**P=**	**0.535**	NS
