# Supplementary material for: Iodixanol Has a Favourable Fibrinolytic Profile Compared to Iohexol in Cardiac Patients Undergoing Elective Angiography: A Double-Blind, Randomized, Parallel Group Study
Source: PLoS One. 2016 Jan 19;11(1):e0147196. doi: 10.1371/journal.pone.0147196 (PMC4718690; doi:10.1371/journal.pone.0147196)
Supplement: S5 Table — (PDF) [file pone.0147196.s007.pdf]

# S5 Table

**Fig 5A**

| | EPR Sinal intensity (AU) | | | | | | | | | | | |
|---|---|---|---|---|---|---|---|---|---|---|---|---|
| | iohexol | | | | | | iodixanol | | | | | |
| time (min) | run 1 | run 2 | run 3 | run 4 | **mean** | **SE** | run1 | run 2 | run 3 | run 4 | **mean** | **SE** |
| 1 | 1400 | 1320 | 1480 | 1800 | **1500** | **74** | 1600 | 1640 | 1560 | 1360 | **1540** | **241** |
| 15 | 9640 | 9360 | 8080 | 7840 | **8730** | **319** | 2920 | 2880 | 2440 | 2280 | **2630** | **1489** |
| 30 | 16640 | 16320 | 15280 | 14640 | **15720** | **327** | 3880 | 3800 | 3240 | 3600 | **3630** | **2803** |
| 45 | 23800 | 22440 | 20920 | 20200 | **21840** | **568** | 5320 | 5240 | 4600 | 4840 | **5000** | **3899** |
| 60 | 30000 | 29920 | 27520 | 26480 | **28480** | **623** | 7000 | 6560 | 5760 | 6080 | **6350** | **5105** |
